# A taxonomic review of the genus *Paracoccidioides*, with focus on the uncultivable species

**DOI:** 10.1371/journal.pntd.0011220

**Published:** 2023-04-27

**Authors:** Raquel Vilela, Sybren de Hoog, Konstanze Bensch, Eduardo Bagagli, Leonel Mendoza

**Affiliations:** 1 Faculty of Pharmacy, Federal University of Minas Gerais, Belo Horizonte, Minas Gerais, Brazil; 2 Microbiology and Molecular Genetics, Biomedical Laboratory Diagnostics, Michigan State University, East Lansing, Michigan, United States of America; 3 Center of Expertise in Mycology of RadboudUMC/Canisius Wilhelmina Hospital, Nijmegen, the Netherlands; 4 Westerdijk Fungal Biodiversity Institute, Utrecht, the Netherlands; 5 Universidade Estadual Paulista, Botucatu, São Paulo, Brazil; Albert Einstein College of Medicine, UNITED STATES

## Abstract

*Paracoccidioides* species have always been surrounded by taxonomic uncertainties. The continuing nomenclatoral muddle was caused in part by the failure of Adolfo Lutz and Jorge Lôbo to name the etiologic agents of human paracoccidioidomycosis and Jorge Lôbo’s diseases, respectively. Early in their history, it was postulated that the cultivable species causing systemic infections belonged in the genus *Paracoccidioides*, whereas the uncultivable species, causing skin disease, were not part of the genus. The taxonomy of these pathogens was further complicated when a similar skin disease with numerous yeast-like cells in infected dolphins was also reported. Due to its phenotypic similarities with that described by Jorge Lôbo in human and its uncultivable nature, it was assumed that the disease in dolphins was caused by the same fungus. Recent molecular and population genetic analysis, however, found the DNA extracted from the uncultivable yeast-like cells affecting dolphins shared common phylogenetic traits with cultivable *Paracoccidioides* species. The study revealed that the uncultivable pathogens comprised 2 different *Paracoccidioides* species, now known as *P*. *ceti* and *P*. *loboi*, correspondingly. To validate *P*. *loboi* binomial, a comprehensive historical critical review of Jorge Lôbo etiology was performed. This review showed the proposed binomial *P*. *loboi* was previously used, and, thus, a replacement name is introduced, *Paracoccidioides lobogeorgii* nom. nov. In addition, in this review, several cultivable human *Paracoccidioides* species are validated, and the generic type species, *P*. *brasiliensis*, is neotypified as the original material could not be traced.

## Introduction

**Fig 1:**
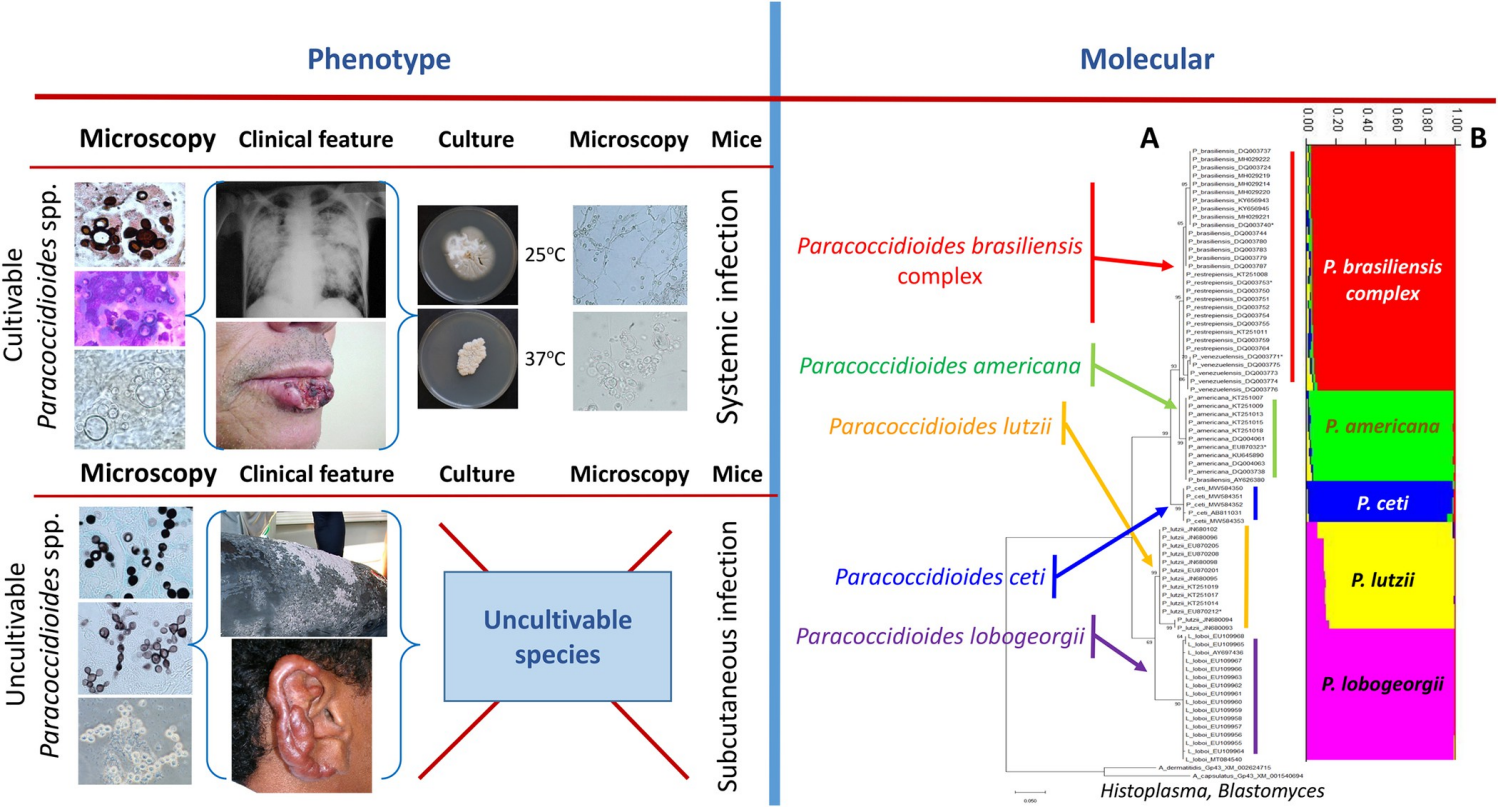
Phenotypic and phylogenetic traits of *Paracoccidioides* species. The figure shows the phenotypic (left) and molecular (right) traits of *Paracoccidioides* species. The phenotypic (microscopy, clinical features, culture, and experimental infection) shows several contrasting differences and few similarities between the cultivable and uncultivable *Paracoccidioides* species. The molecular section depicts an unrooted phylogenetic tree of several *Paracoccidioides* species including *P*. *ceti* from dolphins, *P*. *lobogeorgii*, and 2 dimorphic Onygenales. The tree shows the homologous DNA sequences of the *Gp43* partial coding gene in Maximum Likelihood (**A**) and STRUCTURE (**B**) analyses. With the addition of *P*. *ceti* to the complex, *P*. *lobogeorgii* clustered in a monophyletic group sister to *P*. *lutzii*, whereas *P*. *ceti* formed a monophyletic cluster sister to the cultivable *Paracoccidioides* species (**A**). Population genetics analysis using STRUCTURE 5.2.1 (**A**) [26] recognized 5 populations within the complex (displayed with color bars), supporting the phylogenetic placement of the uncultivable pathogens of human and dolphins within the cultivable *Paracoccidioides* species causing systemic infections.

When Adolfo Lutz [1], in 1908, reported the clinical features of a nasopharyngeal disease in 2 Brazilian human patients, he described its etiology as a fungus developing multiple budding yeast cells in the infected tissues with a mycelial form on various culture media. Based on histopathological preparations, he suggested that the fungus was similar to the one described by Posadas [2] in Argentina (*Coccidioides*) and by Gilchrist [3,4] in the United States of America (*Blastomyces*), but he did not name it. There is no doubt that Lutz’s [1] failure to provide a name for the newly discovered pathogen stirred the many epithets to name its etiologic agent (Table 1). Almeida [5,6], following nomenclatural rules, validly introduced the binomial *Paracoccidioides brasiliensis*. To bring order in the numerous proposed etiologic names, the binomial *P*. *brasiliensis* was officially accepted in 1971, during the first Pan-American Health Organization meeting on paracoccidioidomycosis in Medellin, Colombia [7,8]. With the advent of molecular methodologies, using DNA sequencing, it was found that *P*. *brasiliensis* comprises several cryptic species, and, thus, the species *P*. *lutzii*, *P*. *americana*, *P*. *restrepoana* (*as restrepiensis*), and *P*. *venezuelensis* were introduced (Fig 1) [9–15]. However, some nomenclatural rules to validly publish these species, as required by the code (Arts. 40.7 and 40.8) [16], were not followed.

**Table 1:** *Paracoccidioides brasiliensis* binomials.

Genera and species	Year	Reference
***Zymonema brasiliensis*** *	**1912**	Splendore [17]
***Z*. *histosporocellularis***	**1919**	Haberfeld [18]
***Mycoderma brasiliensis*** *	**1921**	Brumpt [19]
***Monilia brasiliense*** *	**1922**	Vuillemin [20]
***Coccidioides brasiliensis*** *	**1929**	Almeida [5]
***Paracoccidioides brasiliensis***	**1930**	Almeida [6]
***Paracoccidioides cerebriformis*** *	**1935**	Moore [21]
***Proteomyces faverae***	**1935**	Dodge [22]
***Paracoccidioides tenuis*** *	**1938**	Moore [21]
***Coccidioides histosporcellularis***	**1939**	Fonseca [23]
***Lutziomyces histosporcellularis***	**1939**	Fonseca [23]
***Blastomyces brasiliensis*** *	**1942**	Conant and Howell [24]
***Aleurisma brasiliensis*** *	**1951**	Neves and Bogliolo [25]

Binomials used to name the etiologic agent of paracoccidioidomycosis.

*Accepted synonyms of *Paracoccidioides brasiliensis*.

**Fig 2:**
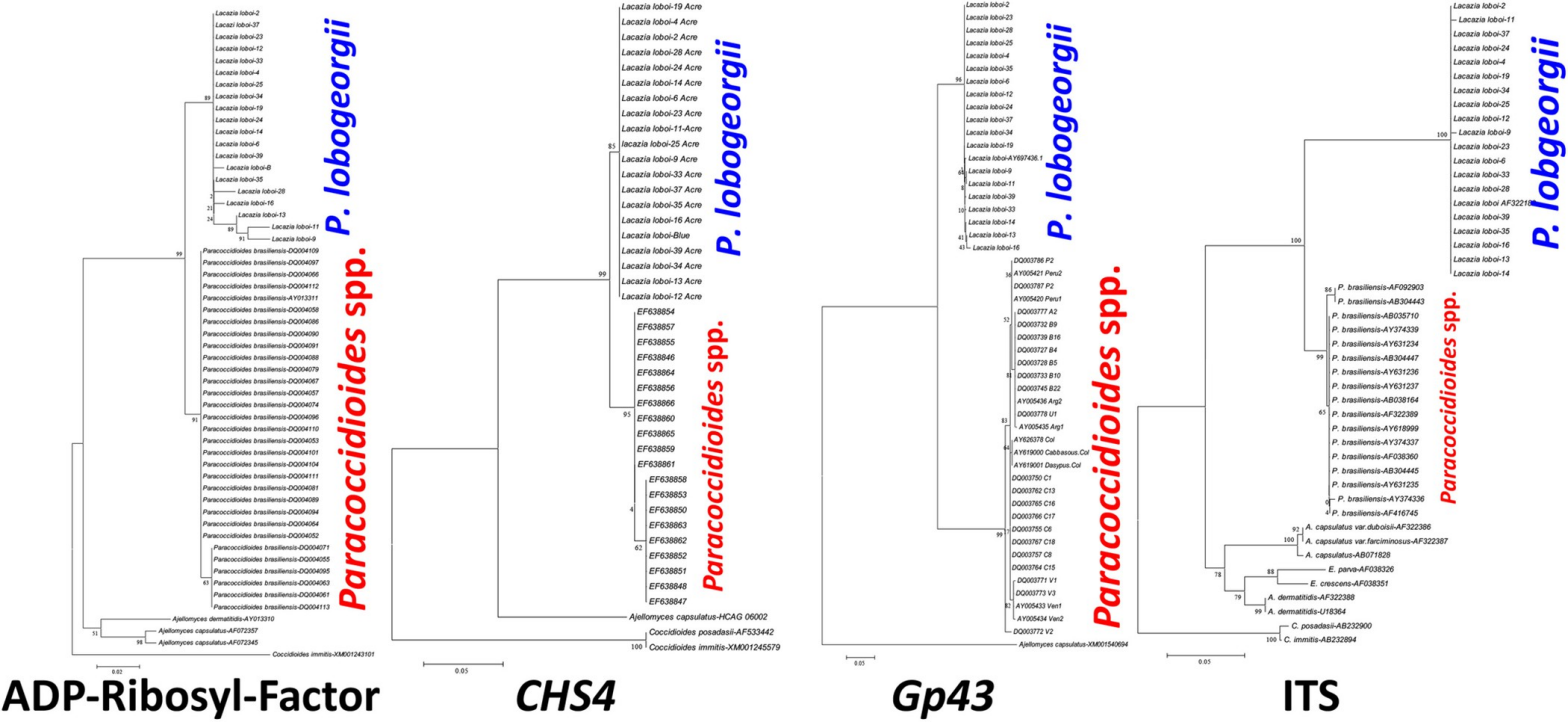
Phylogentic analysis of *Paracoccidioides lobogeorgii* without *P*. *ceti* DNA sequences. Unrooted phylogenetic trees of *Paracoccidioides* species and *P*. *lobogeorgii* (*Lacazia loboi*) without the inclusion of *P*. *ceti*. The figure shows the DNA sequences of *Paracoccidioides* species using *ADP-Ribosylation-factor*, *Chitin synthase 4* (*CHS4*), *Gp43*, Internal Transcriber Spacers (ITS) partial DNA sequences in Maximum Likelihood, and several DNA sequences from dimorphic Onygenales [26]. The figure illustrates the problem faced by earlier investigators conducting phylogenetic analysis without the addition of *P*. *ceti* DNA sequences from dolphins with paracoccidioidomycosis ceti. In the phylogenetic trees, the etiologic agent of Jorge Lôbo’s disease and cultivable *Paracoccidioides* species are each grouped as independent monophyletic clusters.

In a small note, Jorge Lôbo [27] described an infection caused by a fungal yeast pathogen, limited to the subcutaneous tissues, adding more details of the case 1 year later [28]. The disease had histopathological characteristics resembling, somehow, the pathogen reported by Lutz [1]. Judging from the anatomical location of the lesion and the phenotypic traits of the pathogen in the infected tissues, Lôbo believed that he was facing a new type of “blastomycosis,” different to the one reported by Lutz [1] in 1908. Although he described very well the clinical and pathological features of the new entity [29,30], as in Lutz [1] case, he also failed to name the etiologic agent. This omission stemmed a controversy on its etiologic agent that has lasted until the present day [26]. To make things even more complicated for the taxonomy of this pathogen, soon it was found that the yeast-like cells in the host’s infected tissues resisted culturing (Fig 1) [31]. Despite numerous studies confirming its uncultivable nature, some investigators claimed the recovery in culture from patients with Jorge Lôbo’s disease confirmed by histopathology [8,26,31]. However, these fungal isolates were later identified as either common contaminants, or as a *P*. *brasiliensis* isolate (Tables 2 and 3) [26,31]. The latter probably concerned a mislabel mistake from the culture collection, where the isolate had been kept [31]. Oddly, in 1971, Migaki [44] reported similar yeast-like cells in the skin of an infected dolphin dwelling the USA coasts. Based on the clinical and histopathological phenotypic features and its uncultivable nature, it was believed that the dolphin skin disease was caused by the same fungus reported by Jorge Lôbo in humans. Several other dolphin cases were soon diagnosed supporting the concept that Jorge Lôbo’s disease occurs in both humans and dolphins [45]. Early molecular analysis of DNA extracted from of the yeast-like cells from human cases of Jorge Lôbo’s disease showed that the etiologic agent was closely related to *P*. *brasiliensis*, but in its own monophyletic cluster, supporting at that time the proposed genus *Lacazia* (Fig 2) [46–48].

**Table 2:** Jorge Lôbo’s disease etiology through the years.

Genera and species	Year	Reference
***Glenosporella loboi*** *	**1940**	Da Fonseca and Arêa Leão [32]
***Glenosporopsis amazonica***	**1943**	Da Fonseca [33]
***Paracoccidioides loboi*** *	**1948–1949**	Almeida and Lacaz [34]
***Blastomyces loboi***	**1952**	Langeron and Vanbreuseghem [35]
***Loboa loboi*** *	**1956**	Ciferri and colleagues [36]
***Lobomyces loboi***	**1958**	Borelli [37]
***Lacazia loboi*** *	**1999**	Taborda and colleagues [38]
***Candida loboi***	**2015**	Costa [39]

Binomials used to name the etiologic agent of Jorge Lôbo’s disease over the years.

*Accepted synonyms of *Paracoccidioides lobogeorgii*.

**Table 3:** Molecular study of Jorge Lôbo’s disease contaminants suggested as the disease etiology.

Isolate Number	Original Proposed Name	Phenotypic Identification	Molecular Identification	NCBI Accession Numbers
294 [40,41]	None	*Sterigmatomyces holophilus* [41]	*Sterigmatomyces holophilus* [42]	DQ985957
481 [33,41]	*Glenosporopsis Amazonica* [33]	*Aspergillus penicillioides* [40,41]	*Aspergillus penicillioides* [42]	DQ985958
1488 = 525 [33,41]	*Glenosporella loboi* [32] *Loboa loboi* [36]	*Paracoccidioides brasiliensis* [40,41]	*Paracoccidioides brasiliensis* [42]	DQ667982
979 = 852 [40,41]	None	*Aspergillus penicillioides* [40,41]	*Aspergillus penicillioides* [42]	DQ985960
987 = 755 = 767 [40,41]	None	*Aspergillus penicillioides* [40,41]	*Aspergillus penicillioides* [42]	DQ985959
LD1481194 [39]	*Candida loboi* [39]	*Candida* sp. [39]	*Candida tropicalis* [43]	GCA_001005365.1

Original contaminant fungi and *Paracoccidioides brasiliensis* isolates mistakenly identified as the etiologic agents of Jorge Lôbo’s disease, confirmed by molecular methodologies.

The event that changed the way we approach the taxonomy of the genus *Paracoccidioides* occurred when Rotstein and colleagues [49] sequenced the yeast-like cells from a USA infected dolphin. These investigators found that the 28S LSrDNA sequence had high identity with the DNA sequences of *P*. *brasiliensis*. Almost concomitantly, several authors confirmed that the DNA extracted from infected dolphins in Brazil, Cuba, Japan, and the USA [50–54] placed the pathogen of dolphins with the cultivable *Paracoccidioides* species and away from the DNA sequences of the human uncultivable skin pathogen described by Jorge Lôbo. Originally, Vilela and colleagues [55] proposed the name *P*. *brasiliensis* var. *ceti* to differentiate the uncultivable pathogen of dolphins from that of humans. This report was followed by a more comprehensive population genetic analysis of several USA dolphins infected with yeast-like cells of the pathogen [26]. They found that the DNA sequences of the uncultivable pathogen of dolphins and the one reported by Jorge Lôbo were separate species within the genus *Paracoccidioides*. Based on their observations, they introduced the binomials *Paracoccidioides ceti* (due to a misspelling error in the original publication it appears as *P*. *cetii*) [26] for the dolphin pathogen, and *P*. *loboi* for the human pathogen (Fig 1). However, the later binomial had been previously proposed [31], and, therefore, an exhaustive review of the nomenclatural history of Jorge Lôbo’s disease is necessary to investigate this issue.

This study has 2 objectives. First, to critically review previously published literature to investigate the epithets used to describe the etiologic agent of Jorge Lôbo’s disease. Then, if the binomial *P*. *loboi* was validly used, to propose a new name for the Jorge Lôbo’s disease etiology, and second, to evaluate the current taxonomic and nomenclatural status of all *Paracoccidioides* species, to confirm if nomenclatural rules were followed for all proposed names. This is done with the purpose of validating their nomenclatural status if inaccuracies are encountered.

### Critical chronological review of Jorge Lôbo’s disease etiology

Jorge Lôbo, in 1930 [27], reported a new human “blastomycosis,” restricted to subcutaneous tissue and different from the one described 20 years earlier by Adolfo Lutz [1], causing systemic infections in South American patients, which later became known as *Paracoccidioides brasiliensis* [17,56]. Later, Jorge Lôbo [28] published a detailed description of his finding but did not name the etiologic agent. The patient was a 48-year-old Brazilian man that first noted a small nodule on the lumbosacral anatomical region after a trip to the Amazonas, Brazil. The patient referred that the lesions appeared after he was bitten by a snake. Several months later, a small skin lesion appeared at the bite location. The small lesion was highly pruritic and slowly increased in size; thus, it was surgically removed. However, few months later, the lesion reappeared. When the patient came to Lôbo’s attention in Recife, Brazil, he had carried the infection for the last 19 years. The patient was described as a businessperson, but details on other occupational habits were not identified. Histopathological preparation of the infected tissues showed the presence of numerous uniformly in size yeast-like cells in chains, connected by small bridges, different from the yeast cells developed by *P*. *brasiliensis* in infected tissues. Based on this finding, he concluded that it was a new clinical entity, a notion reviewed later in his thesis to become Professor at the Department of Dermatology, Faculty of Medicine Recife, Brazil and in subsequent reviews on the subject [29,30]. Jorge Lôbo inoculated several animals with the yeast-like cells extracted from one of the nodules, without success. He also used *P*. *brasiliensis* antigens in a skin test and in complement fixation assay with negative results. These results confirmed that he was facing a new morbidity [29]. Although Jorge Lôbo inoculated culture media with the yeast-like cells extracted from the lesion in the original patient, he was not able to recover the pathogen in culture [28,29]. Therefore, he remitted his patient to the Institute Oswaldo Cruz, Rio de Janeiro, Brazil.

At Institute Oswaldo Cruz, Rio de Janeiro, Brazil, Olympio da Fonseca junior and A.E. de Arêa Leão [32] collected clinical specimens from the patient remitted by Jorge Lôbo, and a filamentous hyaline fungus was isolated from the biopsied tissues. At room temperature, the fungus grew very slow on Sabouraud dextrose agar, forming white colonies containing septate hyaline hyphae with conidia. The authors considered the isolated fungus as the etiologic agent of the disease. They argued, “*The new forms of reproduction observed in the cultures of this mushroom demonstrate its great affinities with the producing agents of Gilchrist’s disease or North American blastomycosis and South American blastomycosis*.” Based on Nannizzi’s proposal of the new genus, *Glenosporella*, they classified the fungus recovered from Jorge Lôbo’s patient as *Glenosporella loboi* and then deposited a sample at the Oswaldo Cruz Institute’s Fungal Collection, as isolate No 1488. In addition, a subculture of this isolate was also submitted to the Tropical Medicine Institute, Faculty of Medicine, São Paulo University, as isolate No 525. On page 12 of their proposal, they provided details of the genus and the new species fulfilling nomenclatural rules in place at that time [32].

**Fig 3:**
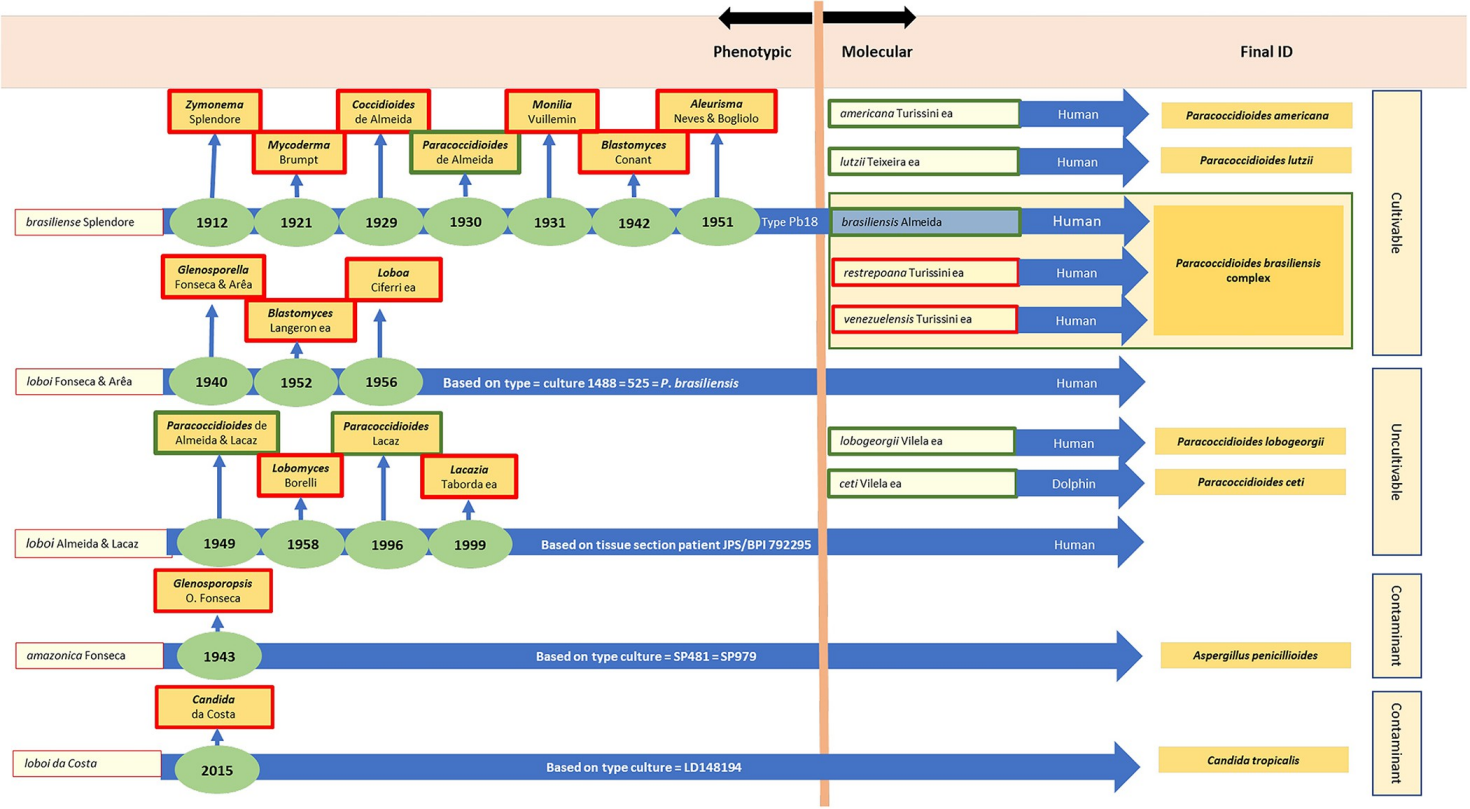
Phenotypic and molecular events in the taxonomy of *Paracoccidioides* species. Phenotypic and molecular features used to justify the different binomials given to the etiologic agents of Lutz’s and Jorge Lôbo’s diseases involving cultivable and uncultivable *Paracoccidioides* species. The left chart depicts the synonyms utilized across the years for both diseases, giving the names and type culture used to justify their names (for *P*. *lobogeorgii* usually contaminant fungi), as well as the years when the binomials were first introduced. The current accepted taxon for the etiologic agents of both diseases is shown within green rectangles, whereas invalid names appeared within red rectangles as per Vilela and colleagues [26].

As previously mentioned, *G*. *loboi* isolate No 1488 was identified later as a *P*. *brasiliensis* isolate; therefore, a theory was proposed to explain the meaning of tube No 1488 containing a typical *P*. *brasiliensis* isolate [31]. Because Jorge Lôbo’s disease agent cannot be cultured, thus the original isolate recovered by da Fonseca and Arêa Leão [32] probably was an environmental fungal contaminant from the patient skin, as was the norm in subsequent cases [31]. Most likely, the original environmental fungus was mistakenly labelled with a previous *P*. *brasiliensis* isolate in the Oswaldo Cruz Institute’s collection, an error common in large culture collections. The binominal *G*. *loboi* proposed by da Fonseca and Arêa Leão [32] (MB#286651) was validly published based on the isolate No 1488 = 525 recovered from the original human case diagnosed by Jorge Lôbo. The authors provided a Latin description and deposited a “type” isolate at the Institute Oswaldo Cruz, Rio de Janeiro, Brazil. Since *G*. *loboi* was later identified as a typical *P*. *brasiliensis* isolate [31] (Table 3), it became conspecific with *P*. *brasiliensis* and thus, a heterotypic synonym of the latter species (Fig 3).

In 1943, Olympio da Fonseca [33] published the clinical features of a similar case to that of Jorge Lôbo. According to him, “*a new type of granulomatose blastomicoide*,” clinically different to that reported by Jorge Lôbo, was found, and, therefore, he argued that its etiology should be different. The patient was previously diagnosed by several dermatologists as a “*Lôbo-type of blastomycosis*, *the latter term having been designated the disease described by Jorge Lôbo and whose parasite Arêa Leão and I described under the name of* *Glenosporella* *loboi*. *The clinical and parasitological study of the case in question*, *led us*, *however*, *to different conclusions*.” Da Fonseca [33] described the patient as a 46-year-old man from the state of Alagoas, Brazil, whose injuries started 20 years earlier. The patient did not remember any special event but suggested mosquito or hematophagous arthropods bites as the initial event. After collecting specimens from the new case, da Fonseca [33] isolated a fungus developing white/grey colonies with aerial branching septate hyaline hyphae with spores. The isolate was deposited at the Tropical Medicine Institute, Faculty of Medicine, São Paulo University, as isolates No 481 and No 979. In addition, isolate No 481 was also deposited at the Institute of Tropical Medicine, Faculty of Medicine, University of São Paulo, Brazil. He published the new species, and on page 711, he gave details of the proposed genus and species *Glenosporopsis amazonica* (MB#286652). The proposal, however, lacked a Latin description, which was a requirement of the Code at that time. In the last part of the proposal, he also combined several other names in the new genus *Glenosporopsis* [33], most of which were later judged to concern unrelated fungi [34,57]. Because of the missing Latin description, all names introduced using da Fonseca [33] proposal are considered invalid. Moreover, the isolate was later found to be *A*. *penicillioides*; therefore, da Fonseca’s [33] binomial became conspecific with *A*. *penicillioides* and, thus, a heterotypic synonym of the latter species (Fig 3, Table 3).

Regarding the placement of the genus *Paracoccidioides* as synonym of *Blastomyces*, this proposal was contested by various authors [31] who argued that “*Conant & Howell* [24] *in 1942*, *placed the genus* *Paracoccidioides* *in the synonymy of* *Blastomyces*, *a fact that was rightly contested by others*. *Under such conditions*, *we believe that the genus* *Paracoccidioides* *described by Almeida and Splendore* is *valid and does not violate any rules of botanical nomenclature*.” Moreover, a similar proposal by Langeron and Vanbreuseghem [35] in 1958 reintroduced again the binomial *Blastomyces loboi* (MB#492449), but the proposal is questioned, since the authors were not fully convinced of the name change, “*il semble qu’on doive la nommer* *Blastomyces loboi*” (it seems we should name it *Blastomyces loboi*).

In 1948, Floriano de Almeida and Carlos da Silva Lacaz [34] studied histological preparations of several cases of Jorge Lôbo’s diseases, including the 2 isolates recovered earlier by da Fonseca and Arêa Leão as isolate No 525 of *Glenosporella loboi* [32] and da Fonseca isolate No 979 of *Glenosporopsis amazonica* [33]. They concluded that da Fonseca and Arêa Leão’s [32] *G*. *loboi* in several culture media behaved as *P*. *brasiliensis*, especially at 37°C, and that *Glenosp*. *amazonica* isolated by da Fonseca [33] does not develop at 37°C. Using the data collected from this study, along with their critical evaluation of several histological preparations from previous cases of Jorge Lôbo’s disease, they concluded, “*Mycologically speaking*, *the fungus that causes Jorge Lobo’s blastomycosis is very similar* (in tissue sections) *to* *Paracoccidioides brasiliensis*, *and should*, *in our opinion*, *be included within the same genus—**Paracoccidioides*, *with the species* *loboi*. *In our view*, *there are not enough reasons to place the agent of keloid blastomycosis in a new genus*” (Fig 3). Floriano de Almeida and Carlos da Silva Lacaz [34] proposal was considered valid since a Latin diagnosis is not necessary when publishing a new combination. The basionym name was validly published as indicated in the paper. Interestingly, on page 16, the binominal is not given as *Paracoccidioides loboi* but as “*Paracoccidioides*, spécie *loboi*,” a statement that complicate the legitimacy of the proposal. However, de Almeida and Lacaz [34] proposal of *P*. *loboi* binomial, followed nomenclatural regulations, and, thus, *P*. *loboi* is currently recognized as validly published name (MB#302227).

In 1956, Raffaele Ciferri [36] from the Center for Human and Comparative Medicine, University of Pavia, Italy, along with Paulo Cordeiro de Acevedo, Sylvio Campos, and Luiz Siqueira Carneiro from Pernambuco, Brazil (MB#299852), published a review on the taxonomy of Jorge Lôbo’s etiology using isolate No 525 provided by Dr. Carlos da Silva Lacaz. The isolate was the same identified earlier by da Fonseca and Arêa Leão [32] as *G*. *loboi*. Apparently, the study was based on the concept of “cryptosporulation” introduced earlier by Ciferri [58]. They indicated that the phenotypic features of *Glenosp*. *amazonica* recovered by da Fonseca [33] should be placed in the genus *Aleurisma* as defined by Vuillemin [8]. No further comments were presented of the isolate recovered by the latter author. Ciferri and colleagues [36] proposed the binomial *Loboa loboi* using isolate No 525 (Fig 3). Since *Loboa loboi* was introduced as a combination based on *G*. *loboi*, the name became a heterotypic synonym of *P*. *brasiliensis* as isolate No 525 = 1488 (*G*. *loboi*) [32], proved to be identical with the latter species.

Two years later, in a one-page note, Dante Borelli [37] reviewed the findings of Siqueira Carneiro [59] regarding isolate No 1488 (*G*. *loboi*) recovered by da Fonseca and Arêa Leão [32]. The author stated that the epithet *Glenosp*. *amazonica* proposed by da Fonseca [33] was built on an *Aspergillus penicillioides* isolate and that *G*. *loboi* as the etiologic agent of Jorge Lôbo’s disease described by da Fonseca and Arêa Leão [32] (isolate No 1488) was based on a typical isolate of *P*. *brasiliensis*. The author’s conclusion was that “…..*based in our current knowledge of this disease*, *the author proposes that it* [Jorge Lobo’s disease] *should be provisionally and concisely called lobomycosis*, *and its agent* *Lobomyces*” (Fig 3). The genus *Lobomyces*, however, was proposed without Latin description and thus invalid. Although, 10 years later, Borelli [60] published a comprehensive review on the synonyms used for the etiologic agent of Jorge Lôbo’s disease, the binomial *Lobomyces loboi* appeared throughout the paper, but without Latin description. From this perspective, it is an invalid binomial. Oddly, in MycoBank (MB#493012), it appeared as validly published name, but we could not trace the citation to validate the reference in their web site.

Ozorio José de Meneses Fonseca and Carlos da Silva Lacaz in 1971 [40] published an extensive review on the etiology of Jorge Lôbo’s disease, giving details on the main clinical and histopathological features of the isolates so far studied from Brazilian cases. The study included isolates No 1488 = 525 (da Fonseca and Arêa Leão) [32] and 481 (da Fonseca) [33], biochemical tests, experimental infection in animals, and immunological tests such as immunodiffusion, precipitation test, and complement fixation. As a critique to Ciferri and colleagues [36], Fonseca and Lacaz [50] argued that they never saw “cryptosporulation” in the genus *Paracoccidioides*. Also, these investigators mentioned that in early studies (de Almeida and Lacaz 1948) [34], one of the authors (CSL) stated that the original “….*princeps isolate was probably exchanged at the Instituto Oswaldo Cruz*, *as there is no doubt that the 1488 isolate at this institution fungal collection represents a typical strain of* *P*. *brasiliensis*, *a fungus that has never been isolated from keloid blastomycosis lesions*.” To investigate this possibility, the authors studied putative isolates (likely contaminants) recovered from cases of Jorge Lôbo’s disease, kept at the Fungal Culture Collection of the Tropical Medicine Institute, São Paulo, Brazil.

The study included isolates recovered from Jorge Lôbo’s disease cases, as follows (1) isolate No 294 recovered by Silvio Campos in Pernambuco, Recife, Brazil, (2) isolate No 481 by Olympio da Fonseca [33] as *Glenosp*. *amazonica*, (3) isolate No 525 (a subculture of isolate No 1488 deposited at the Institute Oswaldo Cruz as *G*. *loboi*) isolated by O. da Fonseca and Arêa Leão [32] from the original case published by Jorge Lôbo, (4) isolate No 979 recovered by Siqueira Carneiro [59] in 1952 from the patient A.A.B. in Pernambuco Recife, Brazil, and (5) isolate No 987 = 481 = 979 recovered by Siqueira Carneiro [59] in 1952 from the patient F.V.M. in Pernambuco Recife, Brazil. The experiment results showed that isolates No 294, 481, 979, and 987 did not develop at 37°C and were negative in experimentally infected mice, whereas isolate No 525 grew at 25°C as hyaline septate hyphae with conidia, and at 37°C as multibudding yeast cells. The authors concluded that the isolates that do not developed at 37°C are unlikely the etiologic agent of Jorge Lôbo’s disease. Based on histological examination of several skin specimens from cases of Jorge Lôbo’s disease, they determined that “*Clinically*, *histopathologically*, *and mycologically*, *the fungus that causes Jorge Lôbo’s blastomycosis is very similar to* *P*. *brasiliensis*, *and should*, *as we see it*, *be included within the same genus—**Paracoccidioides*, *with the species* *loboi*.” Interestingly, these authors make the same mistake as Floriano de Almeida and Carlos da Silva Lacaz [34] by failing to write the full binomial as *Paracoccidioides loboi* compromising the legitimacy of their proposed name.

To validate O.J. de M. Fonseca and C. da S. Lacaz [60] *P*. *loboi* epithet, in 1996, Lacaz [31] published the Latin description of the suggested binomial. He argued that the isolates recovered from the original Jorge Lôbo’s case were all later identified as isolates of *P*. *brasiliensis* or *A*. *penicillioides*; therefore, *P*. *loboi* has priority (Fig 3). In addition, he argued that several lines of evidence, including (1) electron microscopic studies, (2) the cell wall polysaccharides and oligosaccharides, (3) antigenic serology, and (4) the fact that *P*. *loboi* resists culturing, suggested *P*. *brasiliensis* and *P*. *loboi*, are 2 different species. He also mentioned that de Almeida and Lacaz [34] in 1948 had submitted several “neotype” tissue sections from a patient with Jorge Lôbo’s disease (J.P.S.) to 9 different herbaria around the globe to support the species “*loboi*.” Lacaz [31] Latin description for the binomial *P*. *loboi* was not necessary as it was introduced as a new combination. Moreover, the basionym name was not directly indicated in the paper, only indirectly by referring to the original authors of *G*. *loboi* [32]. The designated neotype was not Code compliant as a holotype was indicated in the original publication of *G*. *loboi* [32].

Three years later, Taborda and colleagues [38], using several histological preparations from cases of Jorge Lôbo’s disease, stated that, “*Based upon our study of the lectotype (BPI 792295*, *U*.*S*. *National Fungus Collections) and tissue sections from 35 patients living in the Amazon region of Brazil*, *we concluded that no existing genus can accommodate this taxon*. *We propose a new genus and binomial for the obligate pathogen that causes lobomycosis*.” Therefore, they introduced the binomial *Lacazia loboi*, dedicating the genus names to Dr. Carlos da Silva Lacaz for his many contributions on the etiology of Jorge Lôbo’s disease (Fig 3). Although there are several nomenclatural problems with the proposal, the name was validly published. Both an English and Latin description were provided for the new genus *Lacazia*, but Taborda and colleagues [38] published the type species *Lacazia loboi* as a new combination citing the invalid name *Paracoccidioides loboi* (O.J.M. Fonseca et Lacaz) [40] as basionym, but without giving a full and direct reference to the original author and place of publication as required by the Code [16]. Nonetheless, *Lacazia loboi* was validly published under Art. 38.1 of the Code (https://www.iapt-taxon.org/nomen/pages/main/art_38.html). The use of the term “lectotype” to denote what is, in fact, a holotype was corrected under Art. 9.10 of the Shenzhen Code (https://www.iapt-taxon.org/nomen/pages/main/art_9.html).

Almost concomitantly, Herr and colleagues [46], using molecular methodologies, sequenced several protein coding and noncoding genes using the genomic DNA recovered from yeast-like cells of a Brazilian patient with the disease. Phylogenetic analysis of the investigated genes revealed that *L*. *loboi* was a taxon independent from *P*. *brasiliensis*, supporting Taborda and colleagues’ [38] proposal. Based on Herr and colleagues’ [46] phylogenetic results, Vilela and colleagues [42] explored the possibility of isolate No 525, originally identified as *G*. *loboi* by da Fonseca and Arêa Leão [32], and labelled as *P*. *brasiliensis* by traditional methods, being the etiologic agent of Jorge Lôbo’s disease (Table 3). Using molecular tools, 9 isolates previously identified by traditional methods, including No 294 = *Sterigmatomyces halophilus*; No 481 = *Glenosp*. *amazonica*; No 525, and No 1488 *G*. *loboi* [*Loboa loboi*]; 979 and 852 = *Paracoccidioides loboi*, and isolates No 987 = 755 = 767 = *P*. *loboi* were investigated (Table 3). The isolates were recovered from cases of Jorge Lôbo’s disease and kept at the Institute of Tropical Medicine, Faculty of Medicine, University of São Paulo, Brazil. Phylogenetic analysis found that isolate No 294 was indeed the saprophytic yeast *S*. *halophilus*; isolates No 525 and No 1488 were typical *P*. *brasiliensis* isolates; isolates No 481, and 979 = 852 = 987 = 755 = 767 were all identified as *A*. *penicillioides* environmental contaminants (Table 3). This study further validated the genus *Lacazia* proposed by Taborda and colleagues [38]. Immunological and phylogenetic studies, using the Gp43 antigen and well-known conserved DNA coding regions of *P*. *brasiliensis*, further confirmed that the etiologic agent of Jorge Lôbo’s disease was an independent species closely related to cultivable *Paracoccidioides* species (Fig 1) [42,61,62].

Rotstein and colleagues [49] sequenced the 28S LSrDNA of a dolphin with “lobomycosis” captured in the USA coast and found that it possesses 97% identity with the DNA sequences of *P*. *brasiliensis*. This report was followed by other studies linking the dolphin pathogen to *P*. *brasiliensis* rather than to *L*. *loboi* DNA sequences [50–54]. To investigate this inconsistency, Vilela and colleagues [52] sequenced several coding DNA sequences, including the DNA sequences encoding *Gp43*, *Kex*, and *chitin synthase 4*, from the yeast-like cells recovered from 4 infected dolphins. Based upon the analyses, they proposed that the etiology on the skin granulomas in dolphin was caused by a variety of *P*. *brasiliensis* [55]. To validate their studies, they conducted new phylogenetic and population genetic analyses including new DNA sequences from several infected dolphins [26]. Population genetic analysis using STRUCTURE software placed the etiologic agent of dolphin skin granulomas in a monophyletic cluster sister to *P*. *americana* and *L*. *loboi* from humans formed another monophyletic cluster sister to *P*. *lutzii*, and both clusters grouped sister to *P*. *ceti*, *P*. *americana*, and *P*. *brasiliensis* (Fig 3) [26]. They argued that previous phylogenetic analysis, without the inclusion of DNA sequences from the dolphin pathogen, incorrectly placed *P*. *loboi* away from *Paracoccidioides* species. Furthermore, the authors argued that “*The placement of* *P*. *ceti* *sister to* *P*. *americana* *DNA sequences in this study*, *indicates the use of phenotypic or phylogenetic characteristics without the inclusion of anomalous species*, *can lead to inaccuracies in the taxonomic and phylogenetic classification of these type of microbes*.”

### Taxonomy of Jorge Lôbo’s disease etiology

According to the findings in this review [26,31,34,38,42,54,60], a replacement name for the etiologic agent of Jorge Lôbo’s disease is proposed as follows:

***Paracoccidioides lobogeorgii*** Vilela, de Hoog, Bagagli, and Mendoza, **nom. nov.**

MycoBank MB 844093.

Replaced synonym: *Lacazia loboi* Taborda, V.A. Taborda and McGinnis, J. Clin. Microbiol. 37: 2031 (1999) [38], non *Paracoccidioides loboi* (da Fonseca and Leão) F.P. Almeida and C da S Lacaz (1949) [MycoBank MB 302227].

*Holotype*: Brazil, tissue section from a Brazilian man with Jorge Lôbo’s disease, BPI 792295, slide preserved at BPI [38].

*Etymology*: Dedicated to Dr. Jorge Lôbo, who first described the disease in 1931 [28,29].

*Description*: As per Taborda et al. (1999) [38].

*Disease nomenclature*: The disease names retained are Jorge Lôbo’s disease and paracoccidioidomycosis loboi [55]. Names such as lobomycosis, lacaziosis, Lôbo’s disease, and many others based on obsolete epithets are no longer supported [55].

Regarding the current nomenclatural status of cultivable *Paracoccidioides* species, it was found that the proposed species were invalidly published [10,15], as their holotypes were not correctly indicated, as required by Arts 40.7 and 40.8 of the Code [16]. Therefore, the species *P*. *americana* and *P*. *lutzii* are validated here in. For *P*. *brasiliensis*, a neotype is designated. The type material preserved in a metabolically inactive stage has been deposited at the *Instituto Adolfo Lutz (IAL)*, *São Paulo*, *Brazil* (see below).

*Paracoccidioides americana* Vilela, de Hoog, Bagagli and L. Mend., sp. nov. MycoBank MB 846034.

For a detailed description, see Turissini et al., Fungal Genetics and Biology 106: 22 (2017) [15].

Holotype: Brazil, Botucatu, São Paulo State, isolated from a chronic PCM patient, Pb03 preserved in a metabolically inactive state at the *Núcleo de Coleção de Micro-organismos do Instituto Adolfo Lutz (IAL)*, *São Paulo*, *Brazil*, ID number = IAL 9802. Ext-type: Corporación para Investigaciones Biologicas, CIB_Pb03 = B26.

*Paracoccidioides brasiliensis* (Splend.) F.P. Almeida, Anais Fac. Med. Univ. S. Paulo 5: 134 (1930) MycoBank MB 258811.

For a detailed description, see Turissini et al., Fungal Genetics and Biology 106: 22 (2017) [15].

Holotype: not preserved.

Neotype designated here (MBT 10010030): Brazil, Botucatu, São Paulo State, isolated from a chronic PCM patient, Pb18 preserved in a metabolically inactive state at the *Núcleo de Coleção de Micro-organismos do Instituto Adolfo Lutz (IAL)*, *São Paulo*, *Brazil*, ID number = IAL 9803. Ex-type: Corporación para Investigaciones Biologicas, CIB_Pb18 = B17.

*Paracoccidioides lutzii* Vilela, de Hoog, Bagagli and L. Mend., sp nov. MycoBank MB 846033.

For a detailed description, see Teixeira et al., Med Mycol 52: 26 (2014) [10].

Holotype: Brazil, Goiás state, Goiânia, IPTSP, Universidade Federal de Goiás, clinical isolate, 1992, M.R.R. Silva Pb01, preserved in a metabolically inactive state at the *Núcleo de Coleção de Micro-organismos do Instituto Adolfo Lutz (IAL)*, *São Paulo*, *Brazil*, ID number = IAL 9804. Ex-type: American Type Culture Collection, ATCC MYA-826.

## Discussion

As is common with neglected uncultivable pathogens, for the last 91 years, the etiology of Jorge Lôbo’s disease was poorly understood and, therefore, known under numerous binomials and disease names (Table 2) [31,41,42,55,63,64]. The fact that previous investigators studied the pathogen using phenotypic features alone or combined with traditional methodologies steamed an endless controversy on *P*. *ceti and P*. *lobogeorgii* true taxonomic traits [38,54,55,57]. For instance, various authors reported phenotypic differences between these 2 uncultivable etiologies of dolphins and humans [65,66]. Sadly, they based their conclusions on erroneous premises. For example, Hounbold and colleagues [66] reported that the yeast-like cells of *P*. *ceti* were smaller than those of *P*. *lobogeorgii*. Likewise, Taborda and colleagues [65] suggested that *P*. *ceti* possesses melanin in its cell wall, whereas cultivable *Paracoccidioides* species lack this compound. The truth is that *P*. *ceti* and *P*. *lobogeorgii* both display yeast-like cells between 7 and 25 μm in diameter [41,50,51,52,54,55], and melanin is a key virulence factor in all *Paracoccidioides* species [67]. The above examples described the many difficulties earlier investigators faced in understanding the evolutionary traits of these 2 uncultivable pathogens using traditional methodologies.

A significant phenotypic feature of both, *P*. *ceti* and *P*. *lobogeorgii* adding to their taxonomic mystery, is their intractability to be cultured on mycological media (Fig 1) [53,62]. Interestingly, throughout the years, this characteristic has been at the center of the many fungal contaminants recovered as the etiologic agent of Jorge Lôbo’s disease (Table 3) [26,32–34,36,38,40–44,58,68–71]. This trend culminated with a recent report, incriminating *Candida loboi* as the etiologic agent of Jorge Lôbo’s disease (Table 2) (Fig 3) [39]. Remarkably, a subsequent study found that the genome of the proposed *Candida* species as the etiologic agent of Jorge Lôbo’s disease shared 99.9% identity with the genome of *C*. *tropicalis*, and, thus, a yeast contaminant likely presents in the infected tissues of the patient from which the isolate was recovered [43]. These were understandable mistakes since it is now well known that *P*. *lobogeorgii* yeast-like cells in the infected tissue resist culture. Likely, the above authors, with good intentions, believed that the isolation of the pathogen in culture could help to reveal its taxonomic mystery, a belief that has had a negative impact in unveiling its true taxonomic traits [26,41,47,48].

Although molecular studies were the key in solving the taxonomic attributes of the uncultivable *Paracoccidioides* species, it is of importance to understand why early molecular studies placed *P*. *lobogeorgii* away from the cultivable *Paracoccidoides* species [9,47,48]. When Herr and colleagues [46] performed molecular analysis using *P*. *lobogeorgii* 18S SSU rDNA and *CHS2* genes for the first time, their phylogenetic analysis placed this uncultivable pathogen as the sister taxon to *P*. *brasiliensis* (Fig 2). Their data showed that *P*. *lobogeorgii* was “… .*phylogenetically close to but fundamentally different from* *P*. *brasiliensis*.” They also called the attention to the fact that “*The close similarity or identity of the* *P*. *brasiliensis* *sequences makes it very unlikely that the* *L*. *loboi* *sequences are variants of* *P*. *brasiliensis* *and stands in contrast to the large variation between our two L*. *loboi sequences*.” Their 18S SSU rDNA sequences connecting *P*. *lobogeorgii* to *P*. *brasiliensis* with long branches suggested rapid sub-situation in this rDNA sequence, since short branches were observed between *P*. *brasiliensis* and *P*. *lobogeorgii* using coding gene regions [46–48]. This and several other studies comparing the Internal Transcribed Spacer (ITS) sequences of *P*. *lobogeorgii* with those of other dimorphic fungi consistently placed this pathogen with long branches in a monophyletic cluster sister to *Paracoccidioides* species (Fig 2) [9,47,48,64]. As previously mentioned, it was only when *P*. *ceti* DNA sequences were added in phylogenetic analysis, that *Paracoccidioides* species formed monophyletic groups placing the uncultivable species *P*. *lobogeorgii* and *P*. *lutzii*, sister to cultivable species causing systemic infections (Fig 1) [26,52,55,63]. This was a major finding implying that phylogenetic analysis without the inclusion of atypical members could lead to inaccuracies in the taxonomic classification of anomalous microbes in general [26,50,52,55,70].

Our review on the taxonomic traits of the etiologic agent of Jorge Lôbo’s disease introduces the binomial *P*. *lobogeorgii* to end 91 years of taxonomic ambiguities for the etiologic agent of this uncultivable human pathogen. In addition, the cultivable *Paracoccidoides* species, *P*. *americana*, and *P*. *lutzii* are validated here. The holotypes are deposited in a metabolically inactive state at the *Instituto Adolfio Lutz* (IAL, São Paulo, Brazil) (see above), and their ex-types are available in well-known culture collections [10]. As Splendore [56] original material could not be traced, a neotype is designated as a model for *P*. *brasiliensis*.

**Key Learning Points**

- With the addition of uncultivable species, the phylogeny of *Paracoccidioides* species needed a taxonomic revision.
- The study found that the traditional taxonomy of the genus *Paracoccidioides* need to be validated according to current taxonomic rules following the Code.
- According to the data, the etiology of Jorge Lôbo’s disease, now part of the genus *Paracoccidioides*, needs a taxonomic review. Therefore, following nomenclatural rules, a new name is proposed: *Paracoccidioides lobogeorgii*.
- In addition, several cultivable *Paracoccidioides* binomials did not follow nomenclatural rules. They were validated in this study.
- The review solved 92 years of taxonomic uncertainties for the etiologic agent of Jorge Lôbo’s disease.

**Top Five Papers**

1. Vilela R, Huebner M, Vilela C, Vilela G, Pettersen B, Oliveira C, et al. The taxonomy of two uncultivated fungal mammalian pathogens is revealed through phylogeny and population genetic analyses. Sci Rep. 2021;11:18119. doi: 10.1038/s41598-021-97429-7.
2. Teixeira MdeM, Theodoro RC, Oliveira FF, Machado GC, Hahn RC, Bagagli E, et al. *Paracoccidioides lutzii* sp. nov.: biological and clinical implications. Med Mycol. 2014 Jan; 52:19–28. doi: 10.3109/13693786.2013.794311 PMID: 23768243
3. Turissini DA, Gomez OM, Teixeira MM, McEwen JG, Matute DR. Species boundaries in the human pathogen *Paracoccidioides*. Fungal Genet Biol. 2017;106:9–25. doi: 10.1016/j.fgb.2017.05.007 PMID: 28602831
4. Rotstein DS, Burdett LG, McLellan W, SchwackeL, Rwles T, Terio KA, et al. Lobomycosis in offshore bottlenose dolphins (*Tursiops truncatus*), North Carolina. Emerg Infect Dis. 2009;15:588–590.
5. Vilela R, Mendoza L. Paracoccidioidomycosis ceti (Lacaziosis/Lobomycosis) in dolphins. In: Seyedmousavi S, et al. (eds). Emerging and Epizootic Fungal Infections in Animals. Chapter 9, pp 177–196, 2018. doi: 10.1007/978-3-319-72093-7_9
